# 547. Molnupiravir and Nirmatrelvir/Ritonavir Effectiveness in Reducing the Risk for COVID-19 Disease Progression and Death

**DOI:** 10.1093/ofid/ofad500.616

**Published:** 2023-11-27

**Authors:** Dimitrios Paraskevis, Mary Gkova, Kassiani Mellou, Kassiani Mellou, Kassiani Gkolfinopoulou, Evangelia Georgia Kostaki, Stylianos Loukides, Anastasia Kotanidou, Athanasios Skoutelis, Eleftherios Thiraios, George Saroglou, Dimitrios Zografopoulos, Elias Mossialos, Theoklis Zaoutis, Mina Gaga, Anastasia Antoniadou, Sotirios Tsiodras

**Affiliations:** National Public Health Organization (NPHO), Athens, Greece, Athens, Attiki, Greece; National Public Health Organization (NPHO), Athens, Greece, Athens, Attiki, Greece; Hellenic National Public Health Organization, Athens, Attiki, Greece; Hellenic National Public Health Organization, Athens, Attiki, Greece; Hellenic National Public Health Organization, Athens, Attiki, Greece; Department of Hygiene, Epidemiology and Medical Statistics, Medical School, National and Kapodistrian University of Athens, Athens, Greece, Athens, Attiki, Greece; Second Respiratory Medicine Department, "Attikon" University Hospital, Athens Medical School, National and Kapodistian University of Athens, Athens, Greece, Athens, Attiki, Greece; Evangelismos Hospital, Athens, Iraklion, Greece; Second Department of Medicine and Infectious Diseases, HYGEIA Hospital, Greece, Athens, Attiki, Greece; National Agency for Quality Assurance in Health, Greece, Athens, Attiki, Greece; Department of Internal Medicine, H. Dunant Hospital Center, Athens, Greece, Athens, Attiki, Greece; Greek Ministry of Health, Athens, Greece, Athens, Attiki, Greece; Department of Health Policy, London School of Economics and Political Science, London WC2A 2AE, UK and Institute of Global Health Innovation, Imperial College London, London SW7 2AZ, UK, London, England, United Kingdom; Hellenic National Public Health Organization, Athens, Attiki, Greece; 7th Respiratory Medicine Dept, Athens Chest Hospital "Sotiria", Athens, Attiki, Greece; 4th Department of Internal Medicine, University General Hospital Attikon, Medical School, National and Kapodistrian University of Athens, 12462 Athens, Greece., Athens, Attiki, Greece; 4th Department of Internal Medicine, University General Hospital Attikon, Medical School, National and Kapodistrian University of Athens, 12462 Athens, Greece, Athens, Attiki, Greece

## Abstract

**Background:**

Molnupiravir (M) and Nirmatrelvir/Ritonavir (N/R) have been authorized for use in COVID-19 patients at high risk for severe disease. We aimed to evaluate the effectiveness of M and N/R in highly vulnerable SARS-CoV-2 patients using a retrospective cohort study design.

**Methods:**

The study population consisted of SARS-CoV-2 infected non-hospitalized patients in Greece, aged 65 years or older, that received M between February 2 and March 25, 2022 and N/R between March 26 and July 20, 2022. The impact of each drug was determined via comparisons with age-matched control groups of SARS-CoV-2-positive patients who did not receive oral antiviral therapy, using as outcome (i) hospital admission with COVID-19 within 10 days after tested positive, and (ii) death from COVID-19 within 35 days from positive test.

**Results:**

Overall, 4,240 M and 13,861 N/R recipients were enrolled. Administration of M significantly reduced the risk of hospitalization (Odds Ratio [OR] = 0.40, *p* < 0.001) and death (OR = 0.31, *p* < 0.001) among these patients based on data adjusted for age, previous SARS-CoV-2 infection, vaccination status, and time elapsed since the most recent vaccination. The reductions in risk were most profound among elderly patients (≥75 years old) and among those with high levels of drug adherence. Administration of N/R also resulted in significant reductions in the risk of hospitalization (OR = 0.31, p < 0.001) and death (OR = 0.28, p < 0.001). Similar to M, the impact of N/R was more substantial among elderly patients and in those with high levels of drug adherence.

**Conclusion:**

N/R and M use prevented hospital admissions and death in a large real-world study of highly vulnerable patient populations.

**Disclosures:**

**All Authors**: No reported disclosures

